# Dual opposing regulatory axes of EnvZ/OmpR control biofilm-mediated tolerance and OmpF-dependent resistance to *β*-lactams in *Phytobacter diazotrophicus*

**DOI:** 10.3389/fmicb.2026.1928582

**Published:** 2026-10-26

**Authors:** Jiansheng Lin, Wenbiao Chen, Gaoxiong Wang

**Affiliations:** 1 Microbiology Laboratory, Quanzhou Women's and Children's Hospital, Quanzhou, China; 2 Microbiology Laboratory, The Affiliated Women’s and Children's Hospital of Huaqiao University, Quanzhou, China; 3 School of Basic Medicine, Quanzhou Medical College, Quanzhou, China; 4 Research Administration Office, Quanzhou Women's and Children's Hospital, Quanzhou, China

**Keywords:** antibiotic resistance, biofilm, EnvZ/OmpR, *fliC*, *Phytobacter diazotrophicus*

## Abstract

**Background:**

*Phytobacter diazotrophicus* (*P. diazotrophicus*) is an infrequent opportunistic pathogen. Its biofilm-forming potential and the regulatory function of the EnvZ/OmpR two-component system linking biofilm formation and β-lactam resistance have yet to be elucidated. Herein, we confirmed biofilm production in *P. diazotrophicus* and generated *envZ*/*ompR* knockout mutants to dissect the dual regulatory roles of this two-component system.

**Methods:**

In this study, biofilm formation was qualitatively and quantitatively evaluated through direct visual observation, Gram staining, scanning electron microscopy (SEM), and turbidity measurement. The minimum inhibitory concentrations (MICs) of 12 kinds of β-lactam antibiotics were determined using the MH agar plate dilution method. Gene deletion strains were constructed by homologous recombination. Quantitative reverse transcription PCR (qRT-PCR) and electrophoretic mobility shift assays (EMSA) were performed to explore the regulatory mechanism of the EnvZ/OmpR system. In addition, swimming motility assays were used to characterize biofilm-associated motility phenotypes, and ethidium bromide (EB) accumulation assays were applied to assess bacterial outer membrane permeability.

**Results:**

*P. diazotrophicus* formed robust biofilms on coverslips at the air-liquid–solid interface when cultured in BHI medium, whereas biofilm formation was absent in LB medium. Relative to planktonic counterparts, biofilm-embedded cells displayed significantly elevated MICs to piperacillin, carbenicillin, and meropenem, demonstrating that biofilm matrices confer specific tolerance to these three β-lactams. Deletion of either *envZ* or *ompR* completely abolished biofilm formation compared with the wild type. qPCR assays confirmed marked transcriptional repression of *csgD* and *fliC* in the Δ*ompR* mutant. EMSA assays further proved that OmpR directly interacts with the promoters of *fliC and csgD*. Consistent with this regulatory pattern, loss of *fliC* drastically compromised bacterial swimming motility, which is critical for biofilm assembly. In contrast to the biofilm-associated tolerance phenotype, the Δ*envZ* and Δ*ompR* mutants displayed increased resistance to eight other β-lactam agents. The MICs of the Δ*envZ* and Δ*ompR* mutants increased by 2-fold and 4-fold, respectively, for cefotaxime. Their MICs increased by 4-, 4-, 2-, 2-, 4-, 4-, and 2-fold against cefalotin, cefoxitin, cefuroxime, ceftazidime, cefepime, aztreonam, and ertapenem, respectively. Transcriptomic profiling revealed that four outer membrane porin genes (*ompF*, *ompC*, *ompF2*, *phoE*) were significantly downregulated upon *ompR* deletion. Single gene knockout validation showed that only the Δ*ompF* mutant presented elevated MICs (4-, 8-, 4-, 16-, 4-, 2-, 8-, and 8-fold) to the above eight antibiotics; mutants lacking *ompC*, *ompF2*, or *phoE* exhibited no change in antimicrobial resistance. Additional EMSA results verified direct binding of OmpR to the promoter regions of *ompF*. EB accumulation assays revealed drastically reduced fluorescence intensity in the Δ*ompF* mutant versus the wild type, confirming that OmpF governs outer membrane permeability.

**Conclusion:**

This study reveals a dual antagonistic regulatory mechanism of the EnvZ/OmpR system in modulating β-lactam resistance in clinical *P. diazotrophicus*. EnvZ/OmpR promotes biofilm formation via the *csgD*-*fliC* axis to enhance tolerance to three specific β-lactams, while it upregulates *ompF* to maintain outer membrane permeability and sensitize bacteria to other eight antibiotics. This study first revealed that OmpR directly regulated *fliC* expression. These findings clarify the molecular basis of heterogeneous β-lactam susceptibility and provide novel insights into clinical infection control of this rare pathogen.

## Introduction

*Phytobacter diazotrophicus* (*P. diazotrophicus*) is a Gram-negative bacillus belonging to the Enterobacteriaceae family. In recent years, it has been reported as an opportunistic pathogen in clinical settings (Smits et al., 2022; Lin et al., 2024). The clinical significance of *P. diazotrophicus* has likely been underestimated due to frequent misidentification of clinical isolates as other Enterobacteriaceae (Pillonetto et al., 2018). β-lactam antibiotics are an important means of treating infections caused by *P. diazotrophicus*. There have been reports that multi-drug resistant “superbugs” of *P. diazotrophicus* pose challenges to clinical treatment (Hon et al., 2023; Almuzara et al., 2024).

According to the National Institutes of Health (NIH), approximately 65% of microbial infections and 80% of chronic infections are associated with bacterial biofilms (Muhsin et al., 2015). Since certain pathogenic bacteria tend to form biofilms on the internal and external surfaces of the human body as well as on medical materials, antibiotics are effective in eradicating planktonic bacteria, while bacteria embedded deep inside biofilms are difficult to eliminate. This is one of the reasons why chronic infectious diseases relapse frequently and are difficult to control. Therefore, studying the biofilm and drug resistance mechanisms of *P. diazotrophicus* is of great significance for the prevention and treatment of its infections.

Expression of cellulose and curli fimbriae in *Salmonella typhimurium* relies on the transcriptional regulator CsgD, whose transcriptional activity is modulated by complex nucleoprotein arrangements to govern the transition between planktonic growth and biofilm formation (Gerstel and Römling, 2003). The *fliE* and *fliEFGH* operons for flagellum formation are directly repressed by CsgD in *E. coli*, while CsgD activates the *adrA* gene, which encodes an enzyme for synthesis of cyclic di-GMP, a bacterial second messenger, which in turn inhibits flagellum production and rotation (Ogasawara et al., 2011). The deletion of *fliC* caused the loss of cell motility and the failure to flagella biosynthesis and initiate biofilm formation (Ling et al., 2021).

The two-component system EnvZ/OmpR consists of the sensor histidine kinase EnvZ and the response regulator OmpR, which serves as a canonical signal transduction pathway in bacteria. The *ompR* gene has also been reported to regulate biofilm formation and osmotic stress resistance, and to affect bacterial virulence (Wang et al., 2023). The two-component system EnvZ/OmpR senses fluctuations in Fe^3 + 11^. It modulates biofilm formation via controlling the expression of outer membrane porin gene *ompN*, and also regulates swarming motility as well as type III and type VI secretion systems (Zhang et al., 2025). OmpR promotes biofilm formation by activating the expression of *csgD*, which further induces the synthesis of curli fimbriae that mediate bacterial adhesion (Jubelin et al., 2005; Prigent-Combaret et al., 2001).

In *Escherichia coli* (*E. coli*) K-12, the family of pore-forming proteins includes three members, the OmpF, OmpC and PhoE proteins. These three proteins share a high degree of sequence homology and possess similar β-barrel trimer structural characteristics (Jap and Walian, 1990). This high structural similarity enables all three to act as outer membrane porins, mediating the transmembrane transport of small hydrophilic solutes (such as ions and metabolites) through the formation of transmembrane channels, thus performing the function of a molecular sieve. Meanwhile, the conservation of structure provides a structural basis for their partial functional overlap (e.g., basic substance transport), while differences in sequence and local structure determine their differentiation in specificity (such as ion selectivity and response to environmental regulation) - for instance, PhoE has a preference for anions (Benz et al., 1985). Under high osmotic pressure, OmpC is preferentially expressed, while OmpF dominates under low-osmolarity conditions (Mizuno and Mizushima, 1990). Moreover, both porins contribute to bacterial survival under extremely acidic conditions (Bekhit et al., 2011). The OmpF and OmpC proteins facilitate penetration of β-lactams through the outer membrane (Jaffe et al., 1982). It has been reported that the resistance of *Enterobacter aerogenes* to imipenem is mainly associated with the lack of expression of the homologs of outer membrane proteins OmpC and OmpF, which also results in decreased susceptibility to meropenem and cefepime (Yigit et al., 2002). The downregulation of *phoE* in *Klebsiella pneumoniae* leads to carbapenem resistance (Kaczmarek et al., 2006).

The phosphorylation of OmpR mediated by EnvZ activates the transcription of both outer membrane channel genes *ompF* and *ompC* (Aiba and Mizuno, 1990). Mutions in ompR and envZ result in downregulated expression of outer membrane channel proteins like OmpF and LamB, which reduces outer membrane permeability and decreases the efficiency of antibiotics (such as vancomycin and rifampicin) entering the cells, thereby enhancing bacterial resistance to these antibiotics (Gerken et al., 2024).

In the genome of *P. diazotrophicus* Pd1, apart from the three porin genes *ompF*, *ompC* and *phoE*, gene_3,180 was also annotated as *ompF*. To distinguish these homologous genes, gene_3,180 was designated *ompF2*, and the corresponding protein was named OmpF2,. The biofilm-forming capability of *P. diazotrophicus* and the regulatory roles of the two-component system EnvZ/OmpR in biofilm development and β-lactam resistance remain largely uncharacterized. Herein, we confirmed that clinical *P. diazotrophicus* was capable of forming biofilms at the air-liquid–solid interface. By constructing *envZ* and *ompR* knockout strains and performing phenotypic verification combined with EMSA assays, we demonstrated that EnvZ/OmpR positively regulated biofilm formation through the *csgD*–*fliC* axis, while it modulated bacterial susceptibility to distinct β-lactam antibiotics via controlling *ompF*-dependent outer membrane permeability. This study reveals a novel dual regulatory mechanism underlying heterogeneous β-lactam resistance in *P. diazotrophicus*.

## Materials and methods

### Bacterial strains and culture conditions

Clinical isolate *P*. *diazotrophicus* Pd1, pKAS32 plasmid and the pDM4 plasmid were preserved in our laboratory. *E. coli* BL21 (DE3) and *E. coli* S17-1 (λpir) were purchased from Beyotime Biotechnology. *P*. *diazotrophicus* Pd1 was cultured on LB agar plates.

### Qualitative and quantitative determination of biofilms

Bacteria were resuscitated on LB agar plates for 24 h, after which single colonies was picked and cultured for an additional 24 h. The biofilm-forming ability of *P*. *diazotrophicus* was determined in LB and BHI liquid media (pH 7.2), respectively. The standard protocol of crystal violet semi-quantitative biofilm assay using 96-well flat-bottom polystyrene plates was performed according to according to the reported literature (Kragh et al., 2019).

The biofilms experiment on air-liquid–solid interface of *P*. *diazotrophicus* was carried out following the procedures described below. A sterile coverslip was placed in a 50 mL sterile culture cup, and 12.6 mL of LB or BHI broth was subsequently added to the cup. The turbidity of the bacterial suspension was adjusted to a McFarland standard of 3.0 with a densitometer, then 0.4 mL of the bacterial suspension was transferred into the culture cup, with approximately 5 mm of the coverslip protruding above the liquid surface. After thorough mixing, the culture cup was statically incubated at 37 °C. Biofilms formed on coverslips were subjected to qualitative and quantitative analysis after 24 h and 48 h incubation.

Three approaches, including direct imaging, Gram staining and scanning electron microscopy (SEM), were applied for the qualitative characterization of air-liquid–solid interface biofilms. The coverslips were gently rinsed with phosphate-buffered saline (PBS) to eliminate planktonic bacteria on the surface, followed by direct photography of biofilm bands formed at the air-liquid–solid interface on the coverslips. Subsequently, the biofilm samples were subjected to Gram staining, and the bacterial morphology was observed under an oil immersion objective. Separately, coverslips bearing biofilms were air-dried and observed via SEM at a magnification of 763 × to analyze the overall aggregation morphology of biofilms.

To directly quantify biofilm biomass, biofilm layers were scraped off the coverslips at the air-liquid–solid interface for turbidity measurement. Ten coverslips were cultured in parallel. At 24 h and 48 h post-incubation, biofilms from five coverslips at each time point were harvested by scraping, and the bacterial concentration was determined using a McFarland densitometer. This biological experiment was repeated three times.

### Antibiotic susceptibility test

The minimum inhibitory concentrations (MICs) of the tested strains against 12 β-lactam antibiotics, namely carbenicillin, piperacillin, cefalotin, cefoxitin, cefuroxime, cefotaxime, ceftazidime, cefepime, aztreonam, ertapenem, meropenem and imipenem, were determined via the MH agar plate dilution method. The experimental procedures were carried out in accordance with our previously published literature (Lin et al., 2025b). Briefly, the turbidity of the bacterial suspension was adjusted to the 0.5 McFarland standard with a densitometer, followed by a 100-fold dilution. Afterwards, 2 μL of the diluted bacterial suspension was spotted onto the surface of MH agar plates. All plates were incubated at 37 °C for 20 h before MIC values were recorded. *E. coli* ATCC 8739 was used as the quality control strain, and its MIC test results were within the quality control ranges specified in the Clinical and Laboratory Standards Institute (CLSI) M100-Ed35 document. All biological experiments were conducted in three independent replicates.

### Construction of deletion mutant strains

Gene deletion mutant strains were constructed via homologous recombination according to our previously published report (Lin et al., 2025a). Briefly, the upstream and downstream homologous arms of the *envZ* gene were amplified from the genomic DNA of *P. diazotrophicus* Pd1 by high-fidelity PCR (Vazyme, China) using primer pairs *envZ*-up-F/*envZ*-up-R and *envZ*-down-F/*envZ*-down-R, respectively. The tetracycline resistance gene was amplified from the pKAS32 plasmid using primers *envZ*-TC-F/*envZ*-TC-R. The three amplified fragments were seamlessly ligated into the suicide plasmid pDM4 via Ligation-Independent Cloning (LIC) using the Vazyme MultiS One Step Cloning Kit. The recombinant pDM4 plasmid was electroporated into competent cells of *P. diazotrophicus* Pd1. The successful construction of the *envZ* deletion mutant strain was verified by PCR using the identification primers *envZ*-Delete-F/*envZ*-Delete-R. Similarly, single-gene deletion mutant strains of *ompR*, *csgD*, *fliC*, *ompF*, *ompC*, *ompF2* and *phoE* were constructed following the identical procedure, and all primer sequences are listed in Supplementary Table S1.

### Construction of the complemented strain (Δ*envZ* C-*envZ*)

The shuttle plasmid pBBR1MCS-5 was chosen to construct the *envZ*-complemented strain of the *envZ* knockout mutant. Briefly, The *envZ* promoter and coding sequence were amplified from genomic DNA of *P. diazotrophicus* Pd1 via high-fidelity PCR (Vazyme, China) with primer pairs P*envZ*-F/P*envZ*-R and C*envZ*-F/C*envZ*-R, respectively (Supplementary Table S1). The two amplified fragments were seamlessly ligated into the pBBR1MCS-5 vector double-digested with XbaI and SacI via LIC, using the Vazyme MultiS One Step Cloning Kit. Recombinant plasmid pBBR1MCS-5-*envZ* was obtained via blue-white screening of *E. coli* DH5α transformants. The recombinant pBBR1MCS-5-*envZ* plasmid was electroporated into competent cells of the *envZ*-deletion mutant of *P. diazotrophicus* Pd1. Quantitative reverse transcription PCR (qRT-PCR, see below) was performed using the primer pair *envZ*-Delete-F/*envZ*-Delete-R to detect the mRNA expression level of *envZ* in the Δ*envZ*-complemented strain.

### RNA transcriptome analysis

In this study, RNA transcriptome sequencing analysis was performed on the wild-type strain and *ompR* knockout strain of *P. diazotrophicus* Pd1. A volume of 100 μL overnight bacterial culture was inoculated into 10 mL fresh BHI medium and incubated statically for 24 h. For each strain, 2 mL bacterial suspension was collected and centrifuged at 10,000 rpm for 2 min, followed by three washes with PBS buffer. The prepared samples were sent to Shanghai Majorbio Bio-pharm Technology Co., Ltd. for RNA transcriptome sequencing. Paired-end RNA sequencing of libraries was carried out on the Illumina NovaSeq 6,000 platform. After the read counts of each gene were obtained, DESeq2 software was used to conduct differential gene expression analysis. The screening criteria for differentially expressed genes were set as follows: adjusted *p*-value (padj) < 0.05, with the fold change (FC) of gene expression between samples ≥ 2 for significantly upregulated genes or ≤ 0.5 for significantly.

### RNA isolation and quantitative reverse transcription PCR (qRT-PCR)

qRT-PCR was used to verify the partial transcriptome results. Total RNA was extracted with FreeZol Reagent (Vazyme Biotech Co., Ltd., Nanjing, China), and cDNA was synthesized by reverse transcription using the HiScript III RT SuperMix for qPCR (+gDNA wiper) kit. Quantitative real-time PCR (qPCR) was performed with ChamQ Blue Universal SYBR qPCR Master Mix (Vazyme Biotech) on the Gentier 96 fully automated real-time PCR system (Tianlong Technology Co., Ltd., Xi’an, China). The detailed protocols of the above three experiments were consistent with those previously reported in our published literature (Lin et al., 2025b). Detailed information of all primers is listed in Supplementary Table S1. The housekeeping gene 16S rRNA was selected as the internal reference for data normalization, and the relative expression levels of *csgD*, *hcp*, *fliC* and *rcsA* were calculated via the 2 ^−ΔΔCt^ method. Five biological replicates were included for each group.

### Expression and purification of the recombinant proteins

The coding sequence of *ompR* was amplified by PCR using the genomic DNA of *P*. *diazotrophicus* Pd1 as the template with the primer pair EcoRI-*ompR*-F and XhoI-*ompR*-R. The purified PCR product and pET-24b(+) vector were double-digested with EcoRI and XhoI, followed by ligation of the digested fragments to construct the recombinant plasmid pET-24b-*ompR*. Subsequently, the recombinant plasmid was transformed into *E. coli* BL21 (DE3) competent cells, which was named *E. coli* BL21(DE3) (pET-24b-*ompR*). A single colony of *E. coli* BL21 (DE3) (pET-24b-*ompR*) was inoculated into 500 mL liquid LB medium and cultured with shaking at 37 °C and 220 rpm until the exponential growth phase. IPTG was then added to the bacterial culture to a final concentration of 0.5 mM, and incubation was continued at 37 °C for 3 h to induce protein expression. Purification of the OmpR-His protein was performed as previously described (Lin et al., 2025a). The concentration of the recombinant OmpR protein was determined by a Nano-300 Micro-Spectrophotometer.

The cytoplasmic domain of EnvZ (EnvZc, residues 180–450) was amplified by PCR using genomic DNA of *P*. *diazotrophicus* Pd1 as the template with the primer pairs EcoRI-*envZc*-F/XhoI-*envZc*-R. The purified PCR product and Pet-28a(+) vector were double-digested with EcoRI and XhoI, followed by ligation of the recovered fragments to generate the recombinant plasmid pET-28a-*envZc*. Subsequently, the recombinant plasmid was transformed into *E.coli* BL21 (DE3) competent cells, which was named *E. coli* BL21(DE3) (pET-28a-*envZc*). Induction and purification of the recombinant EnvZc protein were performed as described for OmpR.

### Electrophoresis mobility shift assays

The promoter fragments P*csgD*, P*fliC*, P*ompF* and P*ompF2* were amplified by PCR from the genomic DNA of *P*. *diazotrophicus* strain Pd1 using the primer pairs P*csgD*-F/P*csgD*-R, P*fliC*-F/P*fliC*-R, P*ompF*-F/P*ompF*-R and P*ompF2*-F/P*ompF2*-R, respectively (Supplementary Table S1). The DNA fragment amplified using the primer pair negative-F/negative-R was used as the negative control probe. The concentrations of the five DNA probes were quantified with a Nano-300 micro-spectrophotometer. EMSAs were performed as previously described (Qin et al., 2001), with minor modifications to individually analyze binding of purified OmpR protein to DNA probes of P*csgD*, P*fliC*, P*ompF* and P*ompF2*. The total reaction volume was 20 μL, with final concentrations as follows: OmpR, 1 μM; DNA probe, 16 μM. Binding buffer was used to adjust the volume to 20 μL. Subsequently, the non-denaturing electrophoresis gel was stained with SYBR™ Green for 1 h, and gel images were acquired via a CLINX GenoSens 1,660 imaging system.

For EMSA of partial phosphorylated OmpR, OmpR, EnvZc and DNA probe were pre-mixed in binding buffer at 25 °C for 20 min. The phosphorylation reaction was initiated by adding ATP and MgCl₂, followed by incubation at 25 °C for 5 min. Subsequent electrophoresis and signal detection were performed according to the EMSA procedure described above for unphosphorylated OmpR. The total reaction volume was also 20 μL. The final concentrations of components were as follows: OmpR, 1 μM; EnvZc, 0.5 μM; DNA probe, 16 μM; ATP, 1 mM; MgCl₂, 5 mM. Binding buffer was used to adjust the volume to 20 μL.

### Swimming motility assay

Swimming motility assay was carried out with LB soft agar medium supplemented with 0.3% agar. Single colonies of *P. diazotrophicus* Pd1 wild-type and *fliC* knockout mutant strain were cultured in liquid BHI medium at 37 °C until mid-logarithmic phase. A volume of 1 μL of each bacterial suspension was vertically stabbed into the center of soft agar plates. All plates were incubated upright at 37 °C. The diameters of swimming halos were measured and recorded at 24 h and 48 h post-incubation, and the average values were calculated to assess bacterial swimming motility. Three biological replicates were set for each strain.

### Ethidium bromide accumulation assay

EB accumulation assay was performed to assess bacterial membrane permeability. *P. diazotrophicus* Pd1 Wild-type and *ompF* knockout mutant were cultured in BHI medium at 37 °C to mid-logarithmic phase. Cells were harvested by centrifugation, washed twice with sterile PBS, and resuspended in PBS to an OD₆₀₀ of 0.5. Carbonyl cyanide m-chlorophenyl hydrazone (CCCP) was added to the bacterial suspension at a final concentration of 10 μM, followed by incubation at 37 °C for 30 min, after which EB was supplemented to a final concentration of 2 μg/mL. All samples were incubated at 37 °C in the dark for another 30 min. Fluorescence intensity was measured with a Feyond-F100 microplate reader at an excitation wavelength of 523 nm and an emission wavelength of 564 nm under automatic gain mode, with 30 measurements conducted within 30 min. Relative fluorescence units were recorded to quantify intracellular EB accumulation. Three biological replicates were prepared for each strain.

### Statistical analysis

In the present study, normality of all sample datasets was validated using the Shapiro–Wilk test, while homogeneity of intergroup variances was verified via the Levene’s test. Given these satisfied parametric assumptions, intergroup differences were statistically assessed using Student’s t-test. A *P*-value less than 0.05 was defined as the threshold for statistical significance.

## Results

### *Phytobacter diazotrophicus* can form biofilms at the air-liquid–solid interface on glass slides in BHI

In the preliminary study, our research group found that *P. diazotrophicus* Pd1 barely adheres to polystyrene plates. All bacterial cells were completely washed off after rinsing, producing no crystal violet signal, when cultured in LB and BHI liquid media (pH 7.2) for 24 h and 48 h. Consequently, *P. diazotrophicus* failed to form biofilms on polystyrene plates. However, we unexpectedly found that when statically incubated at 37 °C for 24 h and 48 h, *P. diazotrophicus* formed obvious, thick biofilm at the air-liquid–solid interface on coverslips in BHI liquid medium rather than LB medium (Figure 1). Gram staining revealed that bacteria aggregated into clumps. In addition, SEM revealed that these aggregated bands formed compact clusters with structural characteristics consistent with biofilms (Figure 1). In conclusion, *P. diazotrophicus* is capable of biofilm formation under specific culture conditions.

**Figure 1:**
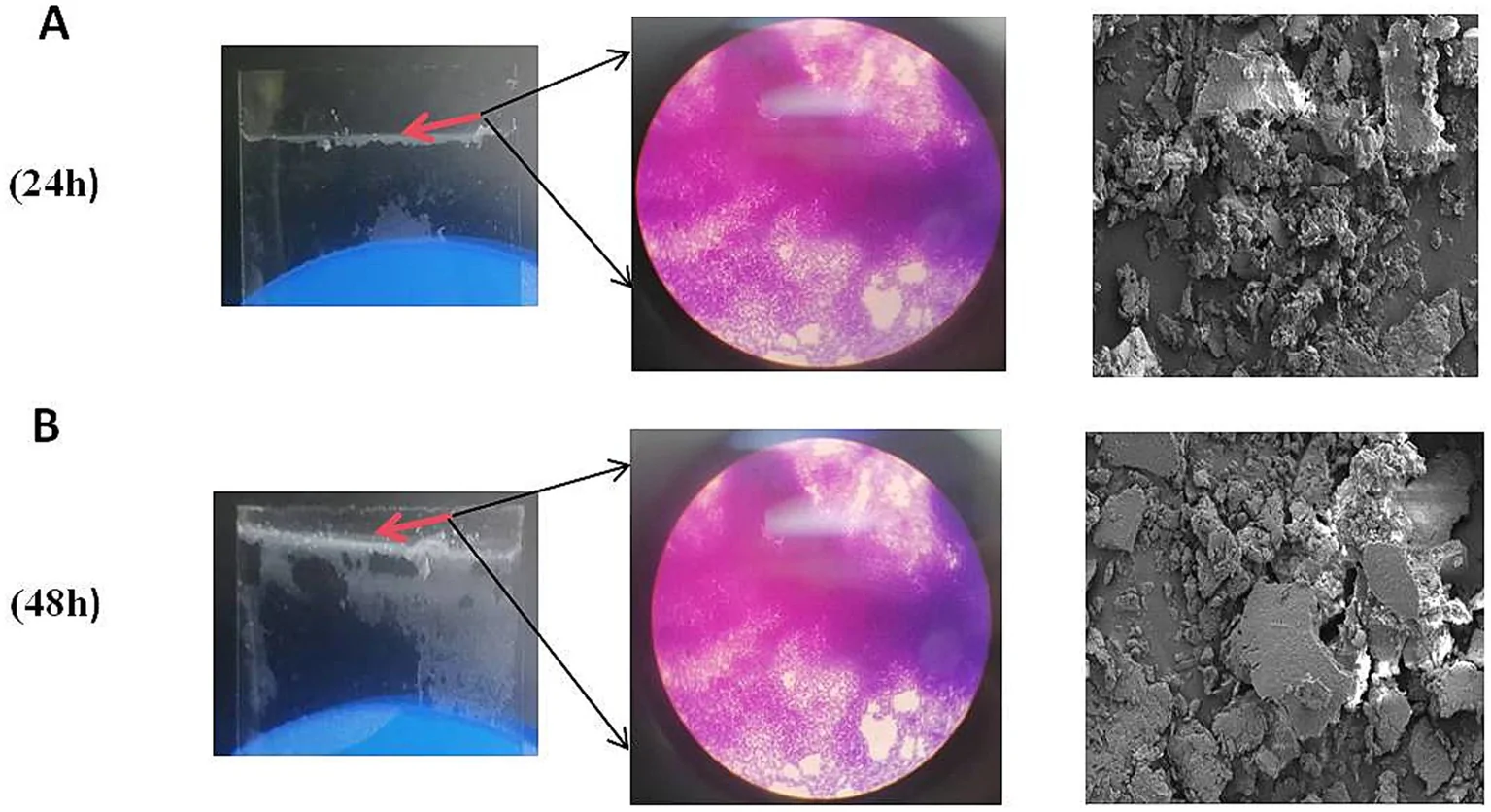
Biofilm formation of *P. diazotrophicus* at the air-liquid–solid interface in BHI liquid media. **(A)** The biofilm formed at the air-liquid–solid interface of the coverslip after 24 h incubation; the left panel shows direct photographic observation of biofilms on the coverslip; the middle panel presents observations of Gram-stained biofilms under an oil immersion microscope; the right panel displays biofilm micrographs obtained by scanning electron microscopy at a magnification of 763×. **(B)** The biofilm formed at the air-liquid–solid interface of the coverslip after 48 h incubation. Similarly, the left panel displays the biofilms on the coverslip captured by direct photography, the middle panel presents the microscopic view of Gram-stained biofilms under an oil immersion objective, and the right panel shows scanning electron micrographs of the biofilms at a magnification of 763 × .

To further verify the correlation between biofilm formation and tolerance to β-lactam antibiotics, the agar dilution method was applied to determine the MICs of antibiotics against planktonic cells and biofilm-associated cells. The MICs of piperacillin were 8 μg/mL for planktonic cells and 32 μg/mL for biofilm cells. The corresponding MICs of carbenicillin were 256 μg/mL and >256 μg/mL, and those of meropenem were 0.0625 μg/mL and 0.25 μg/mL. Compared with planktonic cells, biofilm-associated cells exhibited significantly higher MICs against these three antibiotics, indicating enhanced tolerance. In contrast, no obvious differences in MIC values were observed for the other nine β-lactam antibiotics, demonstrating that biofilms failed to confer protective effects against them. These results further support that the aggregated structure possesses the typical antibiotic tolerance characteristics of bacterial biofilms.

### The EnvZ/OmpR system enhances biofilm formation by positively and directly regulating *csgD* and *fliC*

To clarify the role of the EnvZ/OmpR two-component system in biofilm formation, we constructed *envZ* deletion strain (△*envZ* mutant)and *ompR* deletion strain (△*ompR* mutant). Compared with the wild-type strain, the △*envZ* mutant and △*ompR* mutant failed to form biofilms at the air-liquid–solid interface of glass slides in BHI at 24 h and 48 h (Figures 2A,B). The △*envZ*-complemented strain (Δ*envZ* C-*envZ*) was successfully constructed, and it restored biofilm formation under the same conditions (Figures 2A,B). These results indicate that the EnvZ/OmpR two-component system promotes biofilm formation.

**Figure 2:**
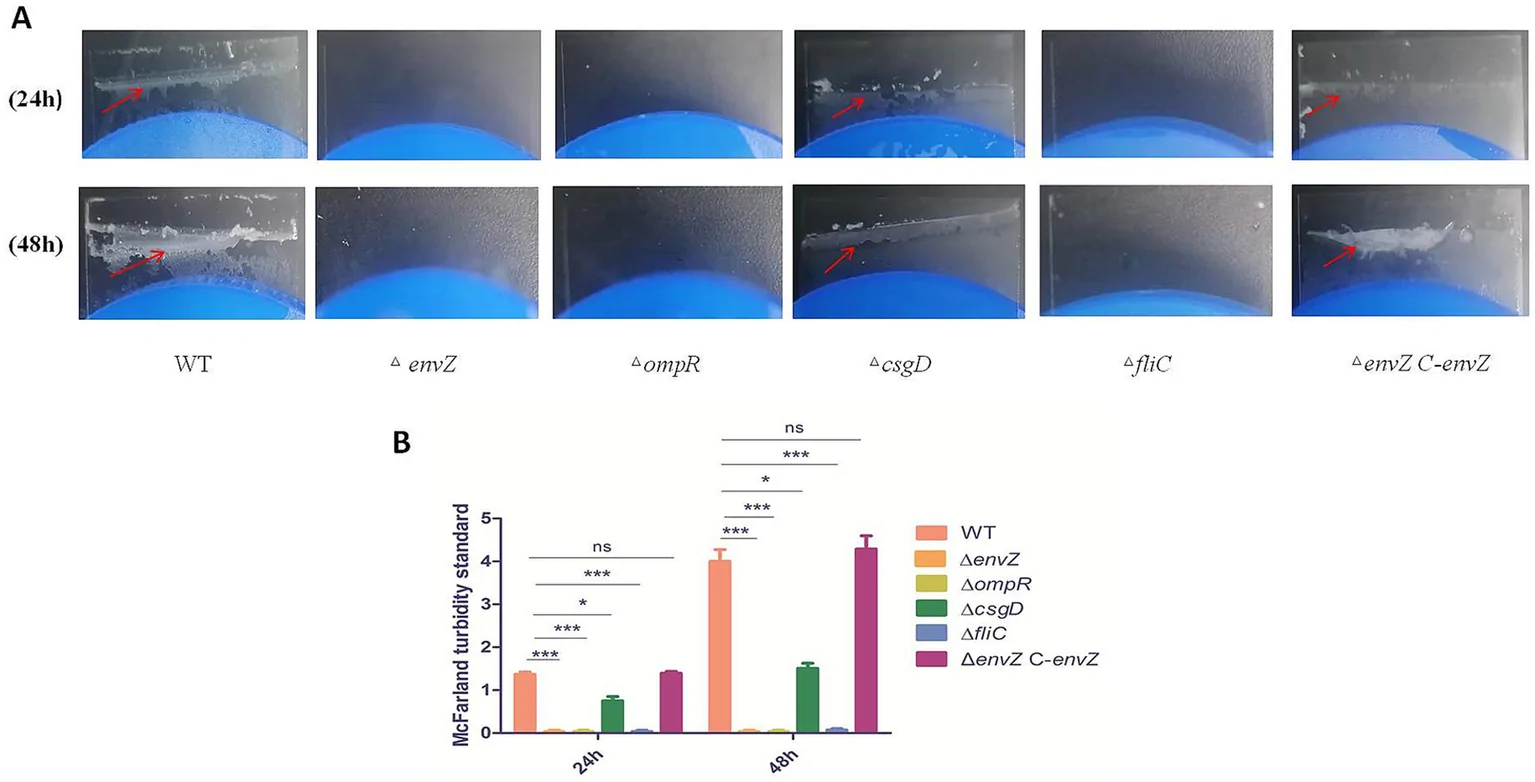
Biofilm formation of different strains for *P. diazotrophicus*. WT indicates the wild-type strain; Δ*envZ*, Δ*ompR*, Δ*csgD*, Δ*fliC* and Δ*envZ* C-*envZ* represent the deletion strains of *envZ*, *ompR*, *csgD*, *fliC* and *envZ* deletion complemented strain, respectively.

To explore the mechanism by which the two-component system EnvZ/OmpR promoted biofilm formation, transcriptomic analysis was performed on the wild-type strain and the △*ompR* mutant. Compared with the wild-type strain, the mRNA expression levels of four biofilm-related genes (*fliC*, *hcp*, *rcsA* and *csgD*) were significantly downregulated in the △*ompR* mutant (Table 1). qRT-PCR verification revealed that only *fliC* and *csgD* were significantly downregulated (Supplementary Figure S1). It is speculated that OmpR/EnvZ regulates these two biofilm genes to facilitate biofilm formation.

**Table 1:** Genes involved in biofilm formation regulated by OmpR.

Gene_id	Gene name	FC (test/control)	*P*_adjust_
Gene_3148	*fliC*	0.359	4.41E-25
Gene_4459	*hcp*	0.359	3.65E-13
Gene_3170	*rcsA*	0.417	0.001070496
Gene_5160	*csgD*	0.388	0.005061861

To determine whether OmpR directly regulates the above two biofilm-related genes, EMSAs were performed. EMSAs results showed that unphosphorylated OmpR and partially phosphorylated OmpR bound to the promoters of *csgD* and *fliC* (Figure 3A). Therefore, OmpR directly regulates *csgD* and *fliC*. However, the shifted band of partially phosphorylated OmpR was not brighter than that of unphosphorylated OmpR (Figure 3A).

**Figure 3:**
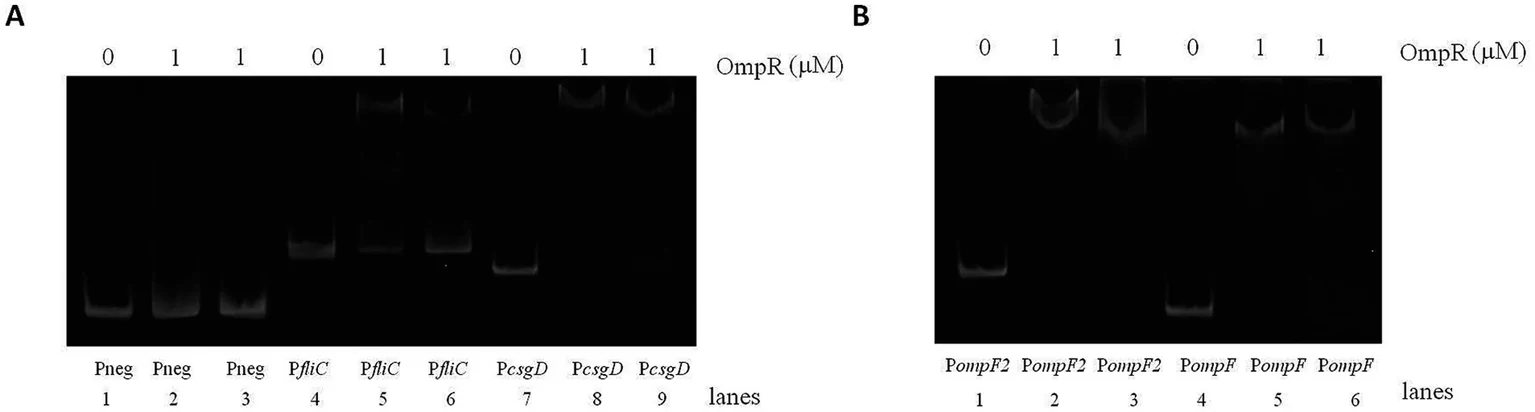
EMSA analysis of the interactions between OmpR and the promoters of two biofilm-related genes (*fliC* and *csgD*), and porin genes (*ompF*and *ompF2*). Note: Pneg, P*fliC*, P*csgD*, P*ompF2* and P*ompF* represent the negative control probe and the promoter regions of the *fliC*, *csgD*, *ompF2* and *ompF* genes, respectively. Lanes 2, 5, and 8 in **(A)** and lanes 2 and 5 in **(B)** represent EMSA results of unphosphorylated OmpR. Lanes 3, 6, and 9 in **(A)** and lanes 3 and 6 in **(B)** show EMSA results after partial OmpR phosphorylation.

To further clarify the roles of *csgD* and *fliC* in biofilm formation of *P. diazotrophicus*, we constructed the *csgD* deletion strain (△*csgD* mutant) and *fliC* deletion strain (△*fliC* mutant). Compared with the wild-type strain, the △*csgD* mutant and △*fliC* mutant exhibited impaired biofilm formation at the air-liquid–solid interface of glass slides at 24 h and 48 h (Figures 2A,B), indicating that *csgD* and *fliC* facilitate biofilm formation. Therefore, the two-component system EnvZ/OmpR promotes biofilm formation by upregulating the expression of *csgD* and *fliC*.

To clarify the specific role of *fliC* in biofilm formation, bacterial swimming motility assays were performed. Compared with the wild-type strain, the swimming ability of the △*fliC* mutant was markedly impaired at 24 h and 48 h (Figure 4). Thus, the EnvZ/OmpR two-component system upregulates *fliC* expression, which strengthens swimming motility and consequently facilitates biofilm formation in *P. diazotrophicus*.

**Figure 4:**
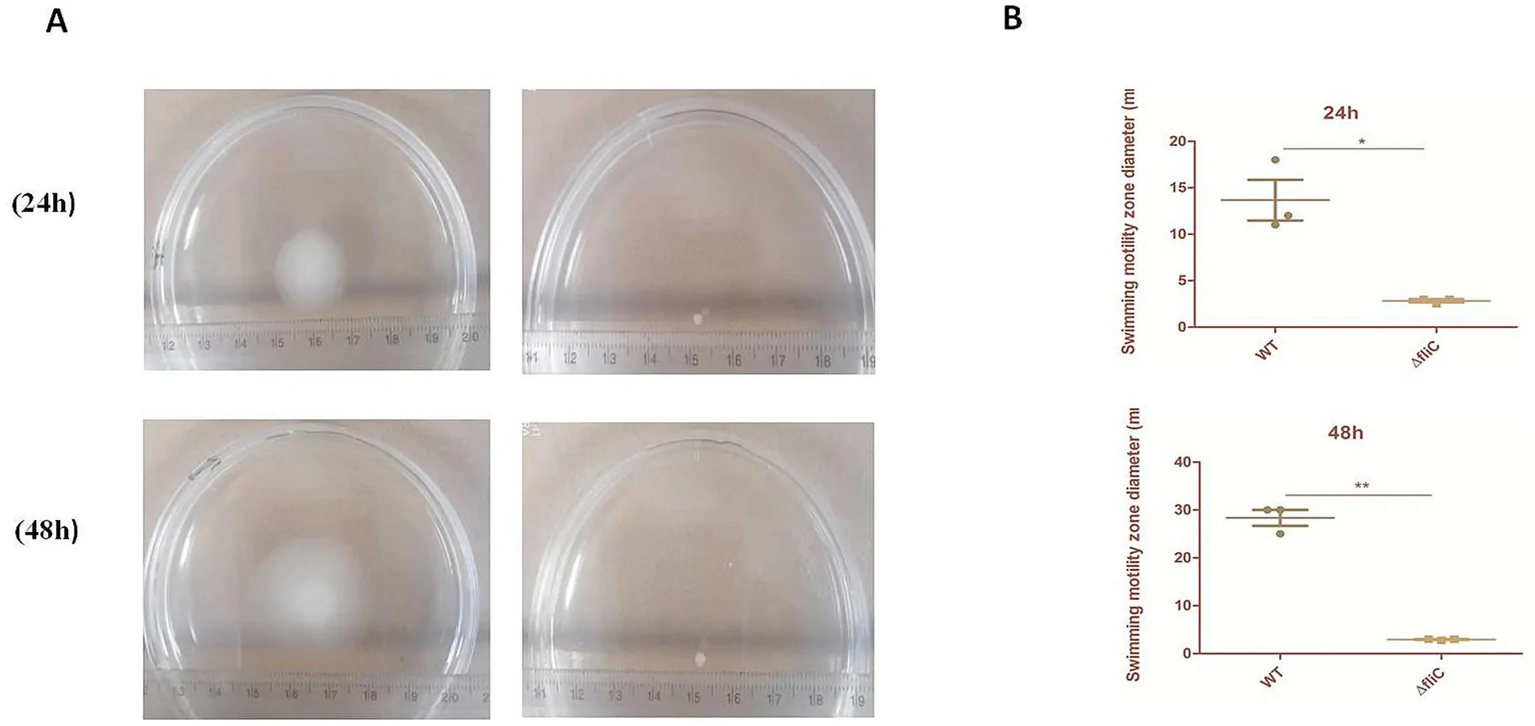
Analysis of swimming motility between wild-type and *fliC* deletion strains of *P. diazotrophicus*. WT and Δ*fliC* represent the wild-type strain and the *fliC* deletion strain, respectively. * and ** indicate *p* < 0.05 and *p* < 0.01, respectively. The smallest graduation of this ruler is 1 mm.

### The two-component system EnvZ/OmpR mediates antibiotic resistance

To clarify the role of the two-component system EnvZ/OmpR in mediating the resistance to β-lactam antibiotics, we performed antibiotic susceptibility tests on the △*envZ* mutant, △*ompR* mutant, and Δ*envZ* C-*envZ*. Compared with wild-type *P. diazotrophicus*, the MICs of the Δ*envZ* and Δ*ompR* mutant increased by 2-fold and 4-fold, respectively, for cefotaxime (Table 2). Their MICs increased by 4-, 4-, 2-, 2-, 4-, 4-, and 2-fold against cefalotin, cefoxitin, cefuroxime, ceftazidime, cefepime, aztreonam, and ertapenem, respectively (Table 2). The Δ*envZ* C-*envZ* strain exhibited MICs restored to the wild-type level for the eight antibiotics described above (Table 2). These findings suggest that the two-component system EnvZ/OmpR reduces resistance to a subset of β-lactam antibiotics.

**Table 2:** The MIC values of antibiotics for wild-type strains and mutant strains.

Antibiotic	MIC (μg/mL)							
	WT	△*ompF*	△*ompC*	△*phoE*	△*ompF2*	△*envZ*	△*ompR*	Δ*envZ* C-*envZ*
Carbenicillin	256.0000	256.0000(0)	256.0000(0)	256.0000(0)	256.0000(0)	256.0000(0)	256.0000(0)	256.0000
Piperacillin	32.0000	32.0000(0)	32.0000(0)	32.0000(0)	32.0000(0)	32.0000(0)	32.0000(0)	32.0000
Cefalothin	4.0000	16.0000(4)	4.0000(0)	4.0000(0)	4.0000(0)	16.0000(4)	16.0000(4)	4.0000
Cefoxitin	8.0000	64.0000(8)	8.0000(0)	8.0000(0)	8.0000(0)	32.0000(4)	32.0000(4)	8.0000
Cefuroxime	4.0000	16.0000(4)	4.0000(0)	4.0000(0)	4.0000(0)	8.0000(2)	8.0000(2)	4.0000
Cefotaxime	0.0156	0.2500(16)	0.0156(0)	0.0156(0)	0.0156(0)	0.0313(2)	0.0625(4)	0.0156
Ceftazidime	0.1250	0.5000(4)	0.1250(0)	0.1250(0)	0.1250(0)	0.2500(2)	0.2500(2)	0.1250
Cefepime	0.0313	0.0625(2)	0.0313(0)	0.0313(0)	0.0313(0)	0.1250(4)	0.1250(4)	0.0313
Aztreonam	0.0313	0.2500(8)	0.0313(0)	0.0313(0)	0.0313(0)	0.1250(4)	0.1250(4)	0.0313
Ertapenem	0.0156	0.1250(8)	0.0156(0)	0.0156(0)	0.0156(0)	0.0313(2)	0.0313(2)	0.0156
Meropenem	0.1250	0.1250(0)	0.1250(0)	0.1250(0)	0.1250(0)	0.1250(0)	0.1250(0)	0.1250
Imipenem	0.2500	0.2500(0)	0.2500(0)	0.2500(0)	0.2500(0)	0.2500(0)	0.2500(0)	0.2500

△ompF,△ompC,△phoE, and △3,180 indicate the ompF, ompC, phoE, and gene_3,180 deletion strains, respectively. The numbers in parentheses represent the fold changes. ΔenvZ C-envZ represents envZ deletion complemented strain.

### The two-component system EnvZ/OmpR increases permeability by upregulating *ompF* expression, thus reducing antibiotic resistance

Transcriptomic results revealed that the expression levels of antibiotic resistance-related genes (*ompF*, *ompC*, *ompF2*, and *phoE*) were downregulated in the △*ompR* mutant (Table 3). This indicates that the two-component system EnvZ/OmpR may modulate these porins to reduce a subset of β-lactam antibiotic resistance.

**Table 3:** Genes involved in antibiotic resistance regulated by OmpR.

Gene_id	Gene name	FC (test/control)	*P*_adjust_
Gene_1865	*ompF*	0.024	0
Gene_0752	*ompC*	0.035	0
Gene_3990	*phoE*	0.49	3.43E-13
Gene_3,180	*OmpF2*	0.453	0.001054725

To further clarify the role of porins in β-lactam antibiotic resistance, we constructed *ompF*, *ompC*, *ompF2* and *phoE* single-gene deletion strains (△*ompF* mutant, △*ompC* mutant, △*ompF2* mutant and △*phoE* mutant) and determined the MICs of 12 β-lactam antibiotics in the wild-type and deletion mutant strains. Compared with wild-type *P. diazotrophicus*, the △*ompF* mutant exhibited 4-, 8-, 4-, 16-, 4-, 2-, 8-, and 8-fold elevated MICs for cefalotin, cefoxitin, cefuroxime, cefotaxime, ceftazidime, cefepime, aztreonam, and ertapenem, respectively (Table 2). However, no obvious changes in antibiotic susceptibility were observed in △*ompC* mutant, △*ompF2* mutant, or △*phoE* mutant. These results demonstrate that the outer membrane protein OmpF, rather than OmpC, OmpF2, and PhoE, is closely associated with β-lactam susceptibility and weakens the resistance of *P. diazotrophicus* to these antibiotics. To clarify whether the two-component system EnvZ/OmpR of *P. diazotrophicus* directly regulates *ompF*, we performed EMSAs. The EMSA results revealed that unphosphorylated OmpR and partially phosphorylated OmpR bound to the promoter region of *ompF* (Figure 3B). Therefore, OmpR directly regulates *ompF* to reduce resistance to a subset of β-lactam antibiotics. In addition, EMSA assays demonstrated that unphosphorylated OmpR and partially phosphorylated OmpR bound to the promoter region of *ompF2* (Figure 3B).

To clarify the effect of *ompF* on the permeability of *P. diazotrophicus*, the fluorescence intensity of EB accumulation was determined in the wild-type strain and the △*ompF* mutant. The △*ompF* mutant exhibited significantly lower EB accumulation fluorescence intensity than the wild-type strain (Figure 5). In summary, the OmpR/EnvZ two-component system upregulates *ompF* expression, enhances cell membrane permeability, and thereby reduces resistance to a subset of β-lactam antibiotics.

**Figure 5:**
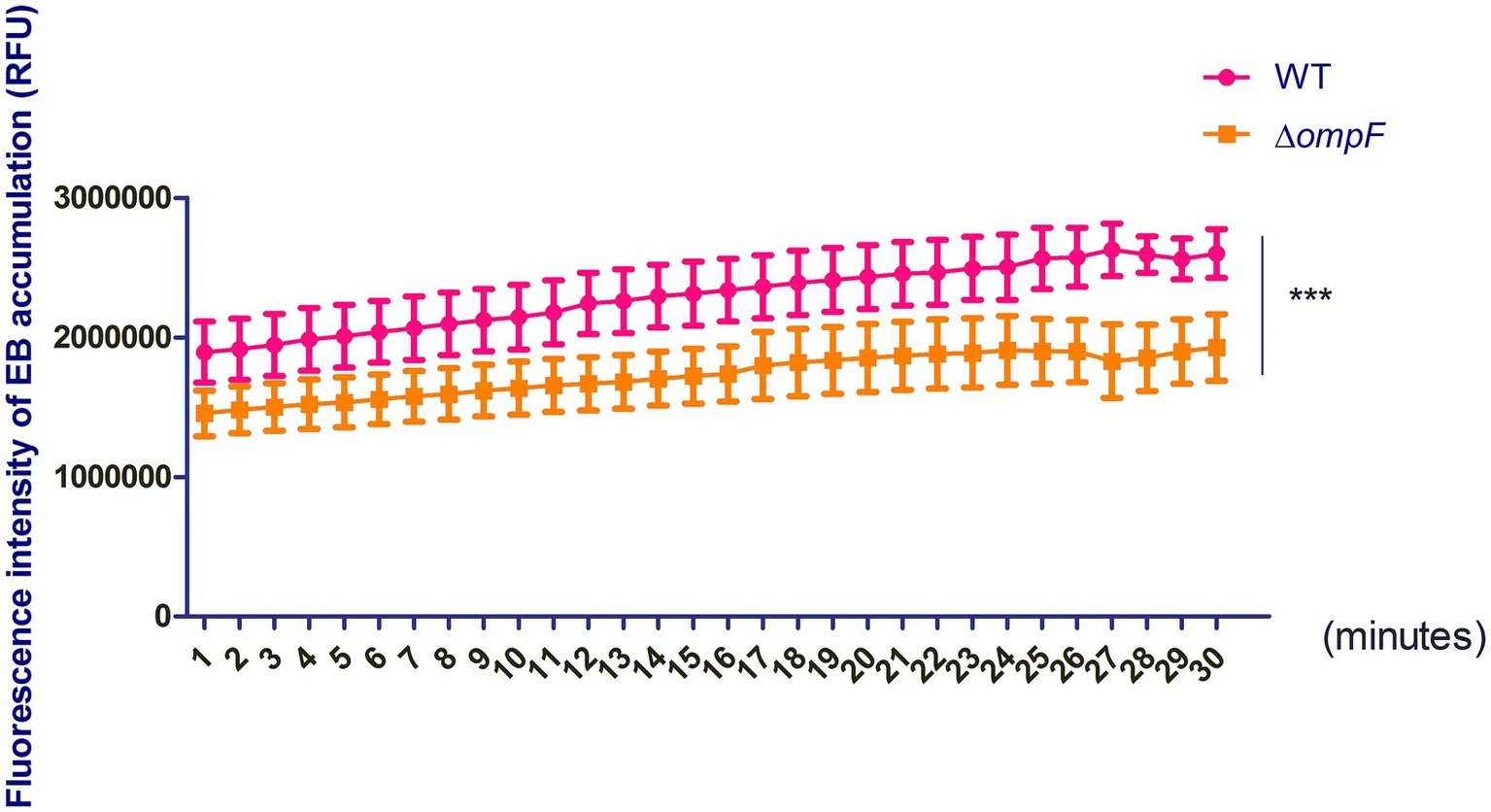
Measurement of EB accumulation fluorescence intensity in wild-type and Δ*ompF* mutant of *P. diazotrophicus* Note: WT and Δ*ompF* represent the wild-type strain and the *ompF* deletion strain, respectively. *** indicate *p* < 0.001.

## Discussion

Previous literature has documented biofilm-forming ability in farmland-derived environmental *Phytobacter* isolates (Nanda et al., 2026), yet the exact species of *Phytobacter* was not specified in that study. To date, there are no clear published reports confirming biofilm formation in *P. diazotrophicus*. Distinct from those environmental strains, the *P. diazotrophicus* strain used in the present study was clinically isolated. While no biofilm was detected for this strain using the polystyrene crystal violet semi-quantitative assay, this study is the first to demonstrate its ability to form biofilms on coverslips at the air-liquid–solid interface. *Pseudomonas fluorescens* (*P. fluorescens*) B52 can form abundant biofilms at the air-liquid–solid three-phase interface on glass coverslips, with the maximum biofilm biomass achieved approximately 20–50 h after inoculation of the liquid medium (Allison et al., 1998). This trend aligns well with our finding that robust biofilm structures of *P. diazotrophicus* emerge at 48 h on coverslips. Unlike *P. fluorescens*, which can form biofilms in polystyrene microplates when cultured in Tryptic Soy Broth supplemented with 0.2% glucose (Rossi et al., 2016), *P. diazotrophicus* in this study failed to produce biofilms on polystyrene microplates. Two explanations may account for this discrepancy. For one, only one clinical isolate was analyzed in this study, and further testing of additional independent strains is required to verify whether this surface-specific biofilm phenotype is widespread among clinical *P. diazotrophicus*. For another, genetic divergence in biofilm regulatory genes may restrict its adhesion to polystyrene, reflecting prominent strain-specific traits. The nutritional composition of culture media serves as a core environmental signal that governs the transition between planktonic and biofilm lifestyles (O'Toole et al., 2000). This readily explains why *P. diazotrophicus* forms biofilms under nutrient-rich BHI conditions rather than in LB medium. Biofilm formation confers strong tolerance to antibiotic therapy, which stems from multiple intrinsic physical barrier mechanisms of biofilms, including massive secretion of extracellular matrix, reduced bacterial growth rates, altered expression of antibiotic targets, and efficient horizontal transfer of resistance genes (Liu et al., 2024). Similarly, the present study found that biofilms formed by *P. diazotrophicus* exhibit tolerance to piperacillin, carbenicillin and meropenem.

Curli are bacterial appendages involved in the adhesion of cells to surfaces; their synthesis is regulated by many genes such as *csgD* and *ompR* (Perni et al., 2016). Six binding sites (D1–D6) for the transcriptional regulator OmpR have been identified on the csgD promoter P*csgD* (Gerstel et al., 2003). OmpR acts as an essential activator to maintain the normal transcriptional expression of P*csgD*; however, phosphorylated OmpR-P represses P*csgD* transcriptional activity upon binding to site D2, whose binding center is located at position −70.5 upstream of D1. The D3–D6 region overlaps with the IHF binding site IHF1. Under microaerophilic conditions, IHF competes with OmpR-P for binding to this region, and this competitive binding mediates the transcriptional response of bacteria to microaerophilic environments (Gerstel et al., 2003). Distinct from this dual regulatory mode in *salmonella*, our EMSA and qRT-PCR data demonstrated that unphosphorylated OmpR of *P. diazotrophicus* specifically binds P*csgD* and upregulates *csgD* transcription. Notably, we did not conduct reporter assays to evaluate the potential repressive activity of phosphorylated OmpR-P. Thus, whether OmpR can exert inhibitory effects on P*csgD* under specific physiological conditions awaits further verification via transcriptional reporter systems. Beyond curli-dependent biofilm assembly, the EnvZ/OmpR system modulates flagellar synthesis and swimming motility in diverse pathogens. In *Yersinia pseudotuberculosis*, OmpR positively regulates the expression of *flhDC* (Hu et al., 2009). Loss of OmpR leads to downregulated transcription of *flhDC* and its downstream flagellar genes such as *fliC*, accompanied by impaired flagellar synthesis and bacterial motility (Hu et al., 2009; Chilcott and Hughes, 2000). However, transcriptomic data from the present study revealed that deletion of *ompR* did not cause significant changes in the expression level of *flhDC* in the strain, suggesting that OmpR regulates *fliC* independent of the *flhDC* pathway in this bacterium. Consistent with our expectation, this study verified the direct and positive regulation of *fliC* expression by OmpR. The apparent inhibitory effect of DNA on the EnvZ phosphatase function is caused by sequestrating OmpR-P from the reaction as a result of OmpR-P binding to DNA (Qin et al., 2001). In this study, we used a strategy wherein wild-type EnvZc allowed partial OmpR phosphorylation to persist after OmpR-P bound to DNA. Nevertheless, the limited phosphorylation level may account for the lack of obvious difference in the shifted bands when unphosphorylated OmpR and partially phosphorylated OmpR bound to the *fliC* promoter. We will further investigate the full phosphorylation of OmpR and its binding sites as our next research direction.

The deletion of OmpF, rather than the deletion of OmpC, leads to increased antibiotic resistance in *Serratia marcescens* (Moya-Torres et al., 2014). This observation is consistent with our results that only the *ompF* knockout strain exhibited increased antibiotic resistance among the four porin-deficient mutants characterized in this work. Genome evolution, and more specifically gene duplication, is a key process shaping host–microorganism interaction. During the evolutionary emergence of the genus *Dickeya*, the gene encoding outer membrane porin OmpF underwent duplication (Cochard et al., 2023). Expression of *ompF2* attenuates the virulence of *Dickeya dadantii*, the causal agent of soft rot disease. The two paralogous copies exhibit distinct expression patterns: *ompF* is constitutively transcribed and can be further activated by the EnvZ-OmpR two-component system, whereas the expression of *ompF2* is tightly regulated (Cochard et al., 2023). Consistent with previous findings in *Dickeya*, two copies of the *ompF* gene were also identified in the genome of *P. diazotrophicus*, both of which were directly and positively regulated by the EnvZ-OmpR system. Nevertheless, this study has certain limitations; the pathogenic role and the detailed regulatory mechanism of *ompF2* in *P. diazotrophicus* remain to be further explored.

Biofilms confer protection via matrix barriers and dormant persister cells. This protective effect only occurs when the antibiotic-targeted PBP functions mainly during rapid bacterial division. Different β-lactams target distinct PBPs, and some agents still inhibit their targets effectively even against slow-growing bacteria, resulting in no obvious biofilm-associated tolerance. The EnvZ/OmpR two-component system exerts opposing regulatory effects on bacterial susceptibility to 11 out of 12 tested β-lactam antibiotics through two separate signaling pathways, and these 11 β-lactam antibiotics fall into two distinct functional groups. Group 1 consists of piperacillin, carbenicillin and meropenem, for which the biofilm matrix acts as the core barrier conferring tolerance. The Δ*ompR* mutant is completely incapable of biofilm formation. While reduced *ompF* expression lowers outer membrane permeability and slightly boosts antibiotic resistance, the absence of biofilm protection dominates the overall phenotype, so the mutant exhibits equivalent resistance to wild-type planktonic cells. Group 2 comprises the remaining eight β-lactams, against which biofilms offer no protective effect; bacterial susceptibility to these antibiotics is primarily governed by outer membrane permeability. *ompR* deletion suppresses *ompF* transcription and reduces intracellular antibiotic accumulation, which drastically enhances mutant resistance to these eight agents. Of note, imipenem, the twelfth β-lactam tested, is unaffected by either biofilm formation or the EnvZ/OmpR regulatory cascade. This unique phenotype may stem from its distinct transmembrane transport characteristics, which require further dedicated research to clarify.

## Conclusion

For the first time, this study verified that *P. diazotrophicus* is able to form biofilms on coverslips at the air-liquid–solid interface in BHI liquid medium. Compared with planktonic cells, biofilm-associated cells exhibited markedly enhanced tolerance to piperacillin, carbenicillin and meropenem. The two-component system EnvZ/OmpR promotes biofilm formation by positively activating the expression of *csgD* and *fliC*. Direct regulation of *fliC* expression by OmpR was identified for the first time in this work. Specifically, *fliC* enhances bacterial swimming motility to facilitate biofilm assembly, which only specifically elevates the strain’s tolerance to the above three antibiotics. Meanwhile, the EnvZ/OmpR signaling cascade upregulates *ompF* expression and increases bacterial outer membrane permeability, rendering the strain more susceptible to most β-lactam antibiotics. Collectively, the EnvZ/OmpR system differentially modulates bacterial resistance to β-lactam antibiotics via two functionally antagonistic pathways. On the one hand, it specifically increases tolerance to piperacillin, carbenicillin and meropenem by inducing biofilm formation; on the other hand, it attenuates resistance to most other β-lactam antibiotics by upregulating OmpF porins and augmenting outer membrane permeability.

## Data Availability

The transcriptome sequencing data are stored in the SRA database of NCBI under the accession number: PRJNA1489329.
