# Supplementary material for: Dual opposing regulatory axes of EnvZ/OmpR control biofilm-mediated tolerance and OmpF-dependent resistance to β-lactams in Phytobacter diazotrophicus
Source: Front Microbiol. 2026 Oct 26;17:1928582. doi: 10.3389/fmicb.2026.1928582 (PMC13760607; doi:10.3389/fmicb.2026.1928582)
Supplement: Supplementary file 1 [file Table_1.doc]

**Table S1 Primer names and sequences in this subject**

| **Primer name** | **Primer sequence(5’-3’)** |
| --- | --- |
| *envZ*-up-F | tgtggaatcccgggagagctcGTGAAGATGGCCTGTCCATCTG |
| *envZ* -up-R | acgacaagcttCCTCATGCTTTAGAACCGTCCG |
| *envZ* -TC-F | agcatgaggAAGCTTGTCGTGTTAAAGACCTCC |
| *envZ* -TC-R | ctgttaAAGCTTTTGGCGGGTGTCG |
| *envZ* -down-F | cccgccaaaagcttTAACAGAAAACCCGCCTTCAGT |
| *envZ* -down-R | cactagtggggcccttctagaCATCGACAACATCGTTAAGCCC |
| *envZ* -Delete-F | CTCACTGGTACCAAGCTCAAGC |
| *envZ* -Delete-R | GATGACGGTCCTGGCATCA |
| *ompR*-up-F | tgtggaatcccgggagagctcCTTTGCCTTCCTGTTGCGG |
| *ompR* -up-R | cacgacaagcttCATTGTTTTTACTCCCAAAGGTTCC |
| *ompR* -TC-F | aaacaatgAAGCTTGTCGTGTTAAAGACCTCC |
| *ompR* -TC-R | tcgcctcatgcAAGCTTTTGGCGGGTGTCG |
| *ompR* -down-F | caaaagcttGCATGAGGCGAATGCGCT |
| *ompR* -down-R | cactagtggggcccttctagaTGTATTCGGATAAACAGCCATGC |
| *ompR* -Delete -F | CGTAGCCCAGACCCCAAAC |
| *ompR* -Delete -R | CGCGCGATAAGCTGATGAA |
| P*envZ*-F | gggggatccactagttctagaTAAGGTGCGGATTAATCATCATTT |
| P*envZ*-R | gcgcattcgcctcatTGTTTTTACTCCCAAAGGTTCCG |
| C*envZ*-F | aaacaATGAGGCGAATGCGCTTCT |
| C*envZ*-F | ctatagggcgaattggagctcGGCAGGAGAAAGCAACGCA |
| q-16S rRNA-F | CAGCCATGCCGCGTGTA |
| q-16S rRNA-R | TAATTCCGATTAACGCTTGCAC |
| q-*csgD*-F | ACTGGCCGCATATCAATGG |
| q-*csgD*-R | ACTCCGAGCTGCTGTAACGAT |
| q-*fliC*-F | CAGAAGTACCTGCCTGTTGCAG |
| q-*fliC*-R | CTCCAACCTGAACAACACCGT |
| q-*hcp*-F | TCTGAAGGTCGATGGCGTTAC |
| q-*hcp*-R | GCGTTTACATGCAGGTCATTG |
| q- *rcsA* -F | TTGGGACTAACGGGGTATCTG |
| q- *rcsA* -R | GCTGACTGCTGACAGGATCGT |
| EcoRI-*ompR*-F | CGG GAATTCGCAAGAGAATTATAAGATTCTGGTAG |
| XhoI-*ompR*-R | CCG CTCGAGTGCTTTAGAACCGTCCGG |
| EcoRI-*envZ*-F | CGG GAATTCCGAATACAGAACCGACCGCT |
| XhoI-*envZ*-R | CCG CTCGAGGTTCTCTTTGCCCGGCGT |
| negative-F | GTGATCGGTAACGCGCACA |
| negative-R | GCCAGTTTCGAGCAGCTAAAG |
| P*csgD*-F | TGTGTGCAAGTATGATTCCGTG |
| P*csgD*-R | ATGAAACCCCGCGTTTAGG |
| P*fliC*-F | GCCCAGCGCAGACTGAGAT |
| P*fliC*-R | AATCGACAGAAAAGCGGGAG |
| P*ompF*-F | TGTGTCTGAGAGGCATCTTTCC |
| P*ompF*-R | CCTGTACAAGATTCACCCTTATCTC |
| P*ompF2*-F | ACCACCCGAATACTAGCGATTC |
| P*ompF2*-R | CGCAGGCACTAAAGTTGCC |
| *csgD*-up-F | tgtggaatcccgggagagctcGAATATTCACACTGTTACAGTCATGCG |
| *csgD* -up-R | cgacaagcttGATGAAACCCCGCGTTTAGG |
| *csgD* -TC-F | gggtttcatcAAGCTTGTCGTGTTAAAGACCTCC |
| *csgD* -TC-R | tcatggcaAAGCTTTTGGCGGGTGTCG |
| *csgD* -down-F | cgccaaaagcttTGCCATGAAACGCGCATC |
| *csgD* -down-R | cactagtggggcccttctagaTCGCTGGTTACCATTCGACC |
| *csgD* -Delete-F | TTGCAGGCAACAGCCTTGT |
| *csgD* -Delete-R | CCCATTGATATGCGGCCA |
| *fliC*-up-F | tgtggaatcccgggagagctcAGCTGCTTTACCCAACGCC |
| *fliC*-up-R | gtctttaacacgacaagcttAATTACTTTCCTTCAAAAATTGATTTAGG |
| *fliC*-TC-F | AAGCTTGTCGTGTTAAAGACCTCC |
| *fliC*-TC-R | taAAGCTTTTGGCGGGTGTCG |
| *fliC*-down-F | gacacccgccaaaagcttTAATACGCTTCAACGTGACATTGA |
| *fliC*-down-R | cactagtggggcccttctagaTTGCTCCAGTCCAAGGTAACG |
| *fliC*-Delete-F | ACAGTACAGACTGCGGAACCTG |
| *fliC*-Delete-R | GCGGCTGGAACAAATACAGC |
| *ompF*-up-F | tgtggaatcccgggagagctcATCCTGGCCCGGTTAACG |
| *ompF*-up-R | gtctttaacacgacaagcttTATTTATTACCCTCATTGGTTTTTTTG |
| *ompF*-TC-F | AAGCTTGTCGTGTTAAAGACCTCC |
| *ompF*-TC-R | ggtgtgctgaAAGCTTTTGGCGGGTGTCG |
| *ompF*-down-F | ccaaaagcttTCAGCACACCTCGTTATTATATGCG |
| *ompF*-down-R | cactagtggggcccttctagaAGGAAATCAGCGGCAACACG |
| *ompF*-Delete-F | TGCTGGTAGCTGGTGCAGC |
| *ompF*-Delete-R | CGTTGTTAACTTTGATACCTGCG |
| *ompC*-up-F | tgtggaatcccgggagagctcTGAATTATCTTTTTGAACTGTACCTGG |
| *ompC*-up-R | cacgacaagcttCATGTTATTAACCCTCGGTTATATGC |
| *ompC*-TC-F | ataacatgAAGCTTGTCGTGTTAAAGACCTCC |
| *ompC*-TC-R | gctgcttaAAGCTTTTGGCGGGTGTCG |
| *ompC*-down-F | cgccaaaagcttTAAGCAGCAATTCTCGACAAAGG |
| *ompC*-down-R | cactagtggggcccttctagaTCCGGCACTTTGTTGGGC |
| *ompC*-Delete-F | GCCCAGGGCAACAACGTT |
| *ompC*-Delete-R | CAAAGCGCAAAACTTCGAAGT |
| *ompF2*-up-F | tgtggaatcccgggagagctcATTCACTTTAAAAATAATTAATCACTGCC |
| *ompF2*-up-R | cacgacaagcttCATAATCTATTCCCTTGAGATCATAATTT |
| *ompF2*-TC-F | agattatgAAGCTTGTCGTGTTAAAGACCTCC |
| *ompF2*-TC-R | tgcttaAAGCTTTTGGCGGGTGTCG |
| *ompF2*-down-F | cccgccaaaagcttTAAGCATACTTTTCTTTCAAAAGCCC |
| *ompF2*-down-R | cactagtggggcccttctagaGCGCATCCAGTTCGAAACC |
| *ompF2*-Delete-F | GGCAACTTTAGTGCCTGCGT |
| *ompF2*-Delete-R | CGACATCATAAGCGACACCATAA |
| *phoE*-up-F | tgtggaatcccgggagagctcGCGGCAAGGAGTTGTTTCG |
| *phoE*-up-R | cacgacaagcttCATTATGTTTCCTGATTAATTGTAATTAACG |
| *phoE*-TC-F | acataatgAAGCTTGTCGTGTTAAAGACCTCC |
| *phoE*-TC-R | gacttaAAGCTTTTGGCGGGTGTCG |
| *phoE*-down-F | cccgccaaaagcttTAAGTCTCGCATAAACGCACTTCA |
| *phoE*-down-R | cactagtggggcccttctagaGGGAGGGTATTATCGCGCC |
| *phoE*-Delete-F | TTTGGCATTAATGATGATCGG |
| *phoE*-Delete-R | TGCTTTCAGG CCAGCGAA |

Notes: Lowercase letters represent overlapping sequences. Underlines indicate restriction enzyme cleavage sites.
